# 223. Clinical Characteristics and Outcomes of *Acinetobacter baumannii* XDR and *Pseudomonas aeruginosa* XDR in a Tertiary Referral Medical Center in Mexico

**DOI:** 10.1093/ofid/ofad500.296

**Published:** 2023-11-27

**Authors:** Alicia Sarahi Ojeda Yuren, Maricarmen Murillo López, Mariana Gómez Abrajan, Gustavo Salinas Arteaga, José Carlos Rodríguez González, María Fernanda Osorno González De León, Sandra Ivet Domínguez Valdez, Araceli Acosta Ruiz, Ana Maria Gónzalez Cardel, Luis Montiel López, Bruno Ali Lopez Luis

**Affiliations:** National Medical Center "20th of November", City of Mexico, Distrito Federal, Mexico; National Medical Center “20th of November”, Mexico City, Distrito Federal, Mexico; National Medical Center “20th of November”, Mexico City, Distrito Federal, Mexico; National Medical Center “20th of November”, Mexico City, Distrito Federal, Mexico; National Medical Center “20th of November”, Mexico City, Distrito Federal, Mexico; National Medical Center, Mexico City, Distrito Federal, Mexico; National Medical Center “20th of November”, Mexico City, Distrito Federal, Mexico; National Medical Center “20th of November”, Mexico City, Distrito Federal, Mexico; National Medical Center “20th of November”, Mexico City, Distrito Federal, Mexico; National Medical Center “20th of November”, Mexico City, Distrito Federal, Mexico; Centro Médico Nacional 20 de Noviembre, Mexico, Distrito Federal, Mexico

## Abstract

**Background:**

Highly resistant gram-negative bacteria such as *Acinetobacter baumannii (AB) Complex* and *Pseudomonas aeruginosa (PA)* have developed into Extremely Drug-Resistant organisms (XDR). This study aims to identify the clinical characteristics and outcomes of bacteremia caused by non-fermenting bacilli (*Acinetobacter baumanni* and *Pseudomonas Aeruginosa*) XDR in a Mexican population of a tertiary medical center in Mexico.

**Methods:**

From January 2019 to December 2021, the results of positive bloodstream cultures for AB XDR and PA XDR were collected from the microbiology laboratory. The electronic records of these patients were obtained, and the variables associated with their clinical characteristics and outcomes were extracted for their analytical description.

**Results:**

A total sample of 49 patients was gathered, from which 26 were men (53%) and 23 (46%) were women, with an average age of 55 years. 71% of patients were admitted to the critical care units. The average Charlson Index was 3.01. 12% had an immunosuppressive condition, 24% had presented a previous hospitalization last year, and 38% had received a previous antibiotic regimen. The length of stay from the AB group had an average of 29 days compared with 46 days in the PA group. The persistence of bacteremia with AB was an average of 11 days versus 22 days with PA. Only 16% of these patients were treated with colistin. The Pitt Index at the time of the first positive culture had an average of 7.56 points in the AB XDR group with 73% mortality, compared to a PA XDR Pitt Index score of 2.4 points and a 42% mortality.

**Conclusion:**

The infections of these pathogens were more prevalent in patients greater than 50 years of age with similar distribution between sexes. Hospitalization in critical care units and immunosuppressive conditions were frequent characteristics of the patients. However, despite the diagnosis of XDR by blood cultures, few patients received colistimethate or another antibiotic with corroborated susceptibility, probably contributing to their complications. Finally, an increase in the length of stay after a positive blood culture is observed in the outcomes, being lower in AB XDR baumannii infections but with higher mortality than PA XDR infections.

**Disclosures:**

**Luis Montiel López, Medical Doctor**, Merck: Speaker|Novartis: Speaker

